# Characterizing Consumer Smartphone Apps for Virtual Reality–Based Exposure Therapy: Content Analysis

**DOI:** 10.2196/41807

**Published:** 2023-04-14

**Authors:** Charvi Sunkara, Rajvi Thakkar, Triton Ong, Brian E Bunnell

**Affiliations:** 1 Psychiatry and Behavioral Neurosciences Morsani College of Medicine University of South Florida Tampa, FL United States; 2 Doxy.me Research Doxy.me Inc Rochester, NY United States

**Keywords:** virtual reality, exposure therapy, phobia, apps, smartphones, VR, smartphone apps, mobile phone apps, content analysis, treatment, clinical evaluation, phobia, consumer apps, mHealth apps

## Abstract

**Background:**

In vivo exposure therapy is the most effective treatment for phobias but is often impractical. Virtual reality exposure therapy (VRET) can help overcome critical barriers to in vivo exposure therapy. However, accessible mobile software related to VRET is not well understood.

**Objective:**

The purpose of our study is to describe the landscape of accessible smartphone apps with potential utility for clinical VRET.

**Methods:**

We conducted a content analysis of publicly available smartphone apps related to virtual reality on the Google Play Store and the Apple App Store as of March 2020.

**Results:**

The initial search yielded 525 apps, with 84 apps (52 on the Google Play Store and 32 on the Apple App Store) included for analysis. The most common phobic stimulus depicted was bodies of water or weather events (25/84, 29.8%), followed by heights (24/84, 28.6%), and animals (23/84, 27.4%). More than half of the apps were visually abstract (39/84, 53.5%). Most apps were free to use (48/84, 57.1%), while the rest were free to try (22/84, 26.2%) or required payment for use (14/84, 16.7%), with the highest cost for use being US $6. The average overall app rating was 2.9 stars out of 5, but the number of ratings ranged from 0 to 49,233. None of the 84 apps advertised compliance with the Health Insurance Portability and Accountability Act, offered the ability to monitor data, provided clinician control over variables in the app experiences, or explicitly stated use by or development with clinicians.

**Conclusions:**

None of the smartphone apps reviewed were explicitly developed for phobia therapy. However, 16 of the 84 included apps were considered ideal candidates to investigate further as part of treatment due to their accessibility, depiction of phobia-relevant stimuli, low or no cost, and high user scores. Most of these apps were visually abstract and free to use, making them accessible and potentially flexible as part of clinical exposure hierarchies. However, none of the apps were designed for clinical use, nor did they provide tools for clinician workflows. Formal evaluation of these accessible smartphone apps is needed to understand the clinical potential of accessible VRET solutions.

## Introduction

Phobias are an intense and pervasive fear of specific objects or situations such as spiders, being in public places, receiving an injection, or seeing blood [1]. Phobias are one of the most common mental health disorders, affecting 1 in 10 adults and 1 in 5 adolescents [2]. Phobia-related avoidance is estimated to cost US $122 billion in lost productivity each year [3]. Individuals with phobias live with greater risks of lower academic achievement, lower socioeconomic status, lower income, employment instability, missed work days, excessive medical services and prescription medications, substance abuse, major depression, and suicide [4,5]. The stigma associated with phobias can lead people to hide their experiences from health care providers, which prevents effective treatment and can result in worsening of phobia symptoms [6]. Phobias can be debilitating with serious impacts on health care systems, the economy, and people’s daily lives. 

Exposure therapy is the most effective evidence-based treatment for phobias [7-9]. During exposure therapy, the individual gradually confronts his or her fear in a controlled environment under the supervision of a therapist, which causes the fear response to weaken over time [10]. Compared to pharmacologic or nonexposure treatments, exposure therapy produces larger and more rapid symptom improvements, causes greater proportions of patients to no longer meet diagnostic criteria, and results in lower rates of relapse [11-13]. Exposure therapy conducted in person, termed in vivo exposure therapy (IVET), is broadly effective but can be stressful, risky, and difficult to implement in office settings [14]. Despite strong evidence supporting exposure therapy for phobias and anxiety disorders, few mental health clinicians use the procedure in practice [15].

Virtual reality (VR) technologies are a promising solution to some critical barriers of IVET. VR-based exposure therapy (VRET) provides immersive simulations that feel real and allow patients to engage with stressful situations safely [16]. Patients accept and engage with VRET more readily than with IVET [17,18]. The computer simulations of VR provide therapists with control and flexibility for the delivery of exposure therapy [19]. VRET treatment effects generalize favorably [20], with treatment outcomes equal to or better than those of IVET [21-23]. The empirical research literature supports VRET for the treatment of phobias and anxiety. However, few sources of information or actionable guidelines exist for the implementation of VRET in clinical practice.

VRET may play an important role in telemedicine in the ongoing COVID-19 pandemic. Social isolation rules and quarantine have led to an increase in phobias [24]. Although VRET is a valuable tool, there are barriers to its implementation, including variability in the methodology of VR studies, questions about the optimal level of graphical realism, and the reproducibility of VR research [25]. Although some hardware and software can be costly to adopt, there are low-cost and portable VR options that involve the use of consumers’ own smartphones, such as Google Cardboard and Samsung Gear VR [21,26]. However, no catalog exists for clinical options among these consumer-oriented apps for VRET. The purpose of this study was to describe the landscape of publicly available smartphone apps with potential clinical utility for VRET. To achieve this purpose, we conducted a content analysis of smartphone apps related to VR on the Google Play Store and the Apple App Store.

## Methods

### Overview

We conducted a content analysis of apps available in the Google Play Store and the Apple App Store to identify and characterize commercially available, consumer-oriented VR smartphone apps that may be used to facilitate VRET for phobias. We focused on the 6 most internationally prominent phobias for this study: animals; blood, injuries, and medical experiences; heights; bodies of water or weather events; enclosed spaces; and flying [2].

### Initial Search

Commercially available apps with potential clinical utility for VRET were identified on the Google Play Store for Android devices and the Apple App Store for iOS devices. Two independent researchers (CS and RT) conducted synchronous searches of both storefronts on March 11, 2020, using the term “virtual reality.” Web Scraper [27] was used to collect app names, descriptions, number of raters, ratings, number of downloads, version, date of latest update, cost, in-app purchases, category, developer name, developer link, age rating, age rating description, language, file size, and age range. The initial search resulted in 525 apps: 295 from the Google Play Store and 230 from the Apple App Store.

### Inclusion Criteria

Apps were included if they (1) were smartphone-based immersive VR apps (ie, intended to be used with a head-mounted display), (2) were available for download in the United States, (3) were available in English, and (4) provided VR content related to the selected phobias: animals; blood, injuries, and medical experiences; heights; bodies of water or weather events; enclosed or confined spaces; or flying. Apps were excluded if they were duplicates within a store, incompatible with the latest Android or iOS operating systems, or could not be operated on study devices.

**Figure 1:**
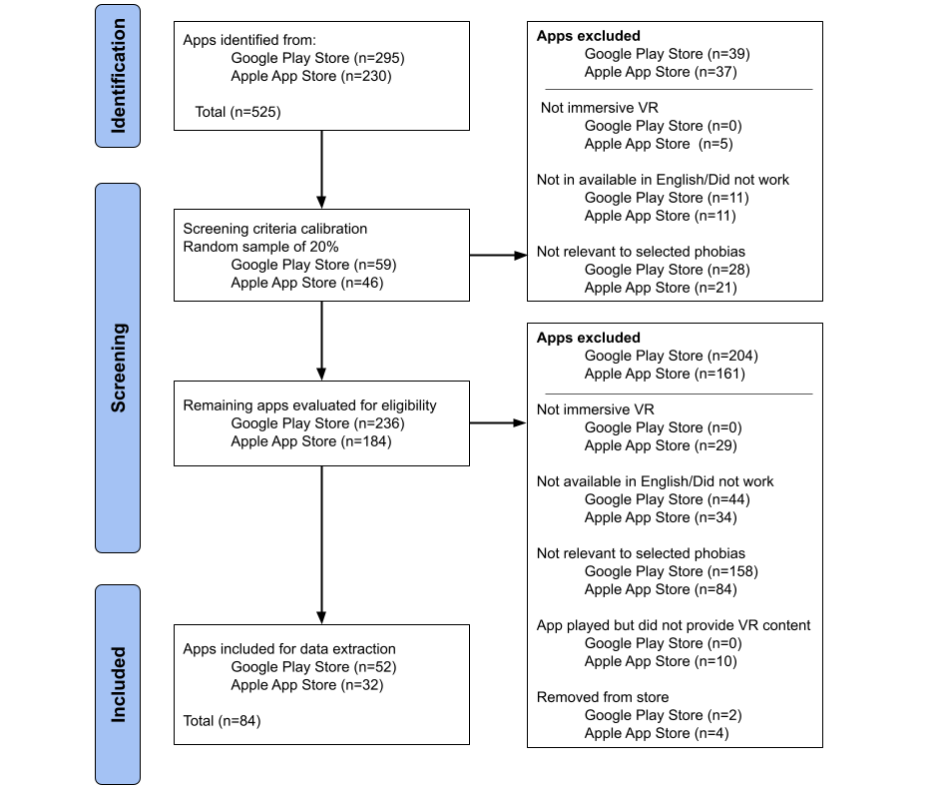
PRISMA (Preferred Reporting Items for Systematic reviews and Meta-Analyses) flowchart for app inclusion. VR: virtual reality.

To calibrate coding of the inclusion criteria, 2 researchers (CS and RT) screened a random sample of 20% of apps from the Google Play Store and the Apple App Store. The overall interrater reliability of coding of the inclusion criteria was 97% for Google Play Store apps and 94% for Apple App Store apps. Disagreements were discussed until consensus was reached, which resulted in the inclusion of 1 additional Apple App Store app. Coders then applied inclusion criteria to the remaining apps from the initial search. This resulted in 54 Google Play Store and 36 Apple App Store apps for inclusion. However, 4 Apple App Store and 2 Google Play Store apps included during initial screening were no longer available on the app stores during data extraction. Therefore, a final total of 52 Google Play Store and 32 Apple App Store apps were included for analysis (Figure 1).

### Data Extraction and Coding

Coding schemes and operational definitions were created and refined, and then the remaining sampled apps were coded independently. Included apps were purchased (if necessary), downloaded, installed, and operated by the researchers to code for potential clinical features. Data extraction included descriptions of app features including the relevant phobia type, level of visual aesthetic, clinician controls, design for therapy, monitoring ability, cost, purchase needs, equipment needed, compliance with the Health Insurance Portability and Accountability Act, and current clinical use. Operational definitions of these codes are provided in Table 1.

**Table 1:** Coding scheme operational definitions.

Code		Operational definition
**Phobia type**		
	Animals	Depicts nonhuman animals (eg, snakes, spiders, and dogs)
	Blood, injuries, or medical experiences	Depicts blood, bodily injury, or medical procedures such as surgery or injection
	Heights	The user could be situated at visibly high locations (eg, looking out from atop a skyscraper, climbing a mountain, or ascending a ladder)
	Bodies of water or weather events	The user could be situated in or near bodies of water or serious weather events (eg, swimming, in the deep sea, or in a heavy storms)
	Enclosed or confined spaces	The user could be situated in a small space with little room to move or escape
	Flying	The user could be inside an airplane or other aircraft
**Visual aesthetic**		
	Abstract	VR^a^ environment was cartoon-like or heavily stylized
	Photorealistic	VR environment was lifelike (eg, a 360° video recording)
	Mix of abstract and photorealistic	VR environment included both stylized and lifelike visuals
**Clinician **		
	Control over variables	The app had features that a clinician could use to control the patient’s VR experience
	No control over variables	No features to control the VR experience
**Designed for therapy**		
	Yes	Apple App Store page included description of explicitly clinical design
	No	No explicit statement of clinical design
**Clinician monitoring capability**		
	Yes	The app had features for a clinician to monitor patient progress
	No	No features for patient monitoring by clinicians
	Cost	The cost of downloading or using the app
**HIPAA^b^ compliance**		
	Yes	App description advertised HIPAA compliance
	No	No explicit advertisement of HIPAA compliance
**Clinical endorsement**		
	Yes	App description includes endorsement or use by clinicians
	No	No explicit endorsement or use by clinicians in the description

^a^VR: virtual reality.

^b^HIPAA: Health Insurance Portability and Accountability Act.

## Results

### App Categorization

The most common phobia stimulus depicted was bodies of water or weather events in 28.8% of Google Play Store apps (15/52), 31.2% of Apple App Store apps (10/32), and 29.8% of all apps (25/84). The second-most common phobic stimulus was heights with 28.8% of Google Play Store apps (15/52), 28.1% of Apple App Store apps (9/32), and 28.6% of all apps (24/84). Animals were the third-most common phobic stimulus with 30.8% of Google Play Store apps (16/52), 21.9% of Apple App Store apps (7/32), and 27.4% of all apps (23/84), as shown in Table 2. Apps could be counted multiple times if they depicted more than 1 phobic stimulus. Apps that depicted numerous phobias in separate simulations were scored as “variety.”

**Table 2:** Apps relating to each phobia.

Phobic stimulus	Google Play Store apps, n	Apple App Store apps, n	Combined apps, n
Water	15	10	25
Heights	15	9	24
Animal	16	7	23
Flying	8	4	12
Enclosed spaces	3	4	7
Variety	4	3	7
Medical	1	1	2

### Level of Realism

Of the apps reviewed in this study, 53.5% (39/84) were visually abstract. An abstract visual style constituted 75.0% (39/52) of Google Play Store apps but only 18.8% (6/32) of Apple App Store apps. As shown in Table 3, there were an equal number of apps in each app store, which had a mix of abstract and photorealistic elements.

**Table 3:** Level of realism.

Level	Google Play Store apps, n	Apple App Store apps, n	Combined apps, n	Examples
Abstract	39	6	45	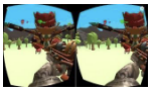 Dinosaur Battle Virtual Reality
Photorealistic	5	18	23	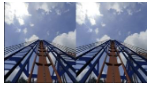 Cedar Point VR
Mixed	8	8	16	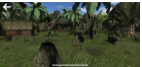 Jurassic Virtual Reality

### Costs

The majority of apps in both the Google Play Store (31/52, 59.6%) and the Apple App Store (17/32, 53.1%) were free to download and use. However, 28.9% (15/52) of Google Play Store apps and 21.9% (7/32) of Apple App Store apps were free to download but required in-app purchases for more advanced features as shown in Table 4. For apps that required payment to download (ie, 6/52, 11.5% of Google Play Store apps and 8/32, 25.0% of Apple App Store apps), the cost ranged from <US $1 to US $5.99. 

**Table 4:** Comparison of free and paid apps.

Cost	Google Play Store apps, n	Apple App Store apps, n	Combined apps, n
Free	31	17	48
Free to try or have in-app purchases	15	7	22
Paid	6	8	14

### User Ratings of the Apps

**Figure 2:**
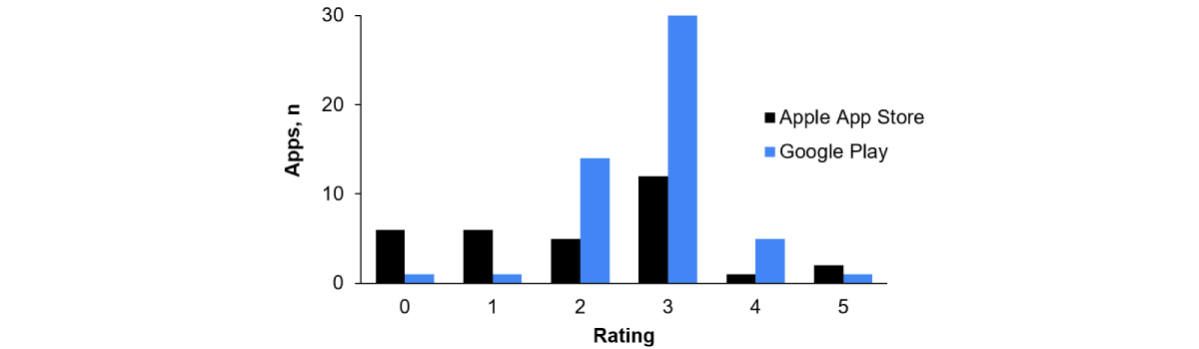
Rating distributions for each app store.

Apps were rated on a scale of 1 to 5 stars (Figure 2). The number of ratings varied substantially among apps (see Table 5), and the distribution of ratings that the reviewed apps received showed an average score of 3.2 stars out of 5 for Google Play Store apps and 3.0 stars out of 5 for Apple App Store apps (see Table 6). ​​The average number of ratings across app stores was 2.9 but ranged from 0 to 49,233 raters.

**Table 5:** Number of ratings for each app.

	App rating			
	Mean (SD)	Median	Mode	Range
Google Play Store apps	3703.3 (8826.03)	742.5	46	0-49,233
Apple App Store apps	1233.83 (4525.5)	20	2	0-21,399
Combined apps	2630.43 (7436.76)	181.5	0	0-49,233

**Table 6:** App score (scores ranging 1-5, with a higher score being better).

	App score		
	Mean (SD)	Median	Mode
Google Play Store apps	3.2 (0.8)	3.1	3
Apple App Store apps	3.0 (1.2)	3.2	3.4
Combined apps	2.9 (1.2)	3.1	3

### Functionality

None of the 84 apps reviewed in the study advertised compliance with the Health Insurance Portability and Accountability Act, offered the ability to monitor data, provided clinician control over variables in the app experiences, or explicitly stated use by or development with clinicians. However, 16 apps depicted stimuli relevant to multiple common phobias, were available for free or at low cost, were available for download at the time of our study, and had a higher average user rating (mean 3.5) compared to the 84 apps overall (mean 2.9; Multimedia Appendix 1). In the Apple App Store, these apps included Aquarium VR, Trail World VR, VR-Virtual Reality Videos, Cedar Point VR, Jurassic Virtual Reality, Roller Coaster VR, Roller Coaster VR Theme Park, and Survival Dino: Virtual Reality. In the Google Play Store, these apps included Amusement Island VR Cardboard, Eagle Survival VR Sim, Shark VR Sharks Games, Underwater Adventure VR, Underwater VR, VR Abyss: Sharks & Sea Worlds in Virtual Reality, VR Diving - Deep Sea Discovery, and VR Ocean Aquarium 3D—all depict bodies of water or weather events and animals, are free to try, have substantial ratings, and most were updated recently at the time of our search. Overall, the Google Play Store had more apps with potential for clinical utility.

## Discussion

### Principal Results

Our study aimed to describe the landscape of consumer-accessible smartphone apps with potential clinical utility for VRET. We conducted a content analysis of smartphone apps related VR on the Google Play Store and the Apple App Store as of March 11, 2020. Of the initial 525 apps, 84 apps (32 on the Apple App Store and 52 on the Google Play Store) were included for data extraction. Bodies of water or weather events (25/84, 29.8%) were the most common phobic stimuli presented, followed by heights (24/84, 28.6%), animals (23/84, 27.4%), flying (12/84, 14.3%), enclosed spaces (7/84, 8.3%), a variety of phobic stimuli (7/84, 8.3%), and medical experiences (2/84, 2.4%). The average app rating was 3.0 (SD 1.2) for Apple App Store apps, 3.2 (SD 0.8) for Google Play Store apps, and 2.9 (SD 1.2) for all apps overall. The number of ratings ranged from 0 to 49,233 raters with an average of 3703.3 (SD 8826.03) on the Google Play Store, 1233.8 (SD 4525.5) on the Apple App Store, and 2630.4 (SD 7436.8) for all apps overall. The majority of the apps were free to use (48/84, 57.1%), while some were free to try (22/84, 26.2%) and few required payment to use (14/84, 16.7%).

These findings are important for identifying easily accessible smartphone VR apps with potential use in clinical VRET. The phobias addressed in this study were bodies of water or weather events, animals, heights, flying, enclosed spaces, and medical experiences. The majority of the apps reviewed are free to use or cost up to a modest US $6. However, it is important to note that while the apps reviewed in this study may have potential clinical utility, there is no direct scientific support for their clinical effectiveness, and none of the apps were designed for clinical use explicitly. While informative, consumer ratings of apps have been based historically on functionality and aesthetic appeal rather than their effectiveness in evoking behavioral change [28-30]. Evaluation of these apps in usability and clinical studies will be an important next step to understanding the value of consumer-oriented apps for therapy.

Most of the apps reviewed in this study were visually abstract (45/84, 53.6%) rather than photorealistic (23/84, 27.4%) or had a mix of abstract and photorealistic elements (16/84, 19.1%). These visually abstract apps may be more appropriate for earlier stages of exposure hierarchies. However, there is ongoing debate on the effect of the visual style on VR-based clinical outcomes. While some studies have found that greater realism in VR correlated with greater immersion and a sense of presence [31,32], others have found that clinical populations preferred simpler and more abstract visuals [33,34]. Future studies should evaluate components of photorealism as they apply to VR experiences and clinical outcomes.

Consumer engagement with the apps in this study varied substantially. For example, the average number of app ratings was 2630.4 but the SD was 7436.8 with a range from 0 to 49,233. From our results, we can understand that user engagement with the apps reviewed contained extremes ranging from apps with no ratings, to 2 ratings for a score of 5.0, to 49,223 ratings resulting in a score of 3.5. Ratings alone should not be considered to assess the clinical potential of an app. The vast majority of mental health app use tends to be isolated to a select few successes [35]. Another study showed that about 75% of patients find and use apps for mental health without input from their mental health provider [36]. These findings offer insights into how complex the process of curation and selection of apps can be for a clinician. Thus, these results may serve as an initial selection of apps to be evaluated further in VRET research.

None of the apps reviewed contained tools for clinicians to control or monitor the patient experience. Although dedicated clinical VRET apps have been developed, such as Amelia Virtual Care and Virtually Better, these apps tend to be proprietary and hardware-bound, restricted for clinic access only, often costly, and do not appear on consumer app stores. The apps reviewed in this study were publicly available on consumer devices, mostly free to use, and potentially useful for expanding clinical options. With some creativity, providers may incorporate consumer-oriented apps into mental health treatment [37]. Future research should aim to assess the clinical effectiveness of these nonclinical apps by using them as clinicians would. As popularity and user ratings may not correspond to clinical utility, such apps need to be evaluated formally to curate content for clinicians [28].

### Strengths

This study was conducted with several key strengths. It is a comprehensive review of accessible VR apps on consumer app stores as of the date of the search (March 2020). Data collection was largely automated and, therefore, many aspects of the content analysis were completed objectively. Analyses involving subjective interpretation had an excellent interrater reliability that was further discussed till a consensus was reached. Furthermore, the apps’ contents were verified via hands-on use of each app for direct assessment of phobia relevance, as opposed to reliance on app marketing material. These methods produced a list of apps that can be further assessed for utility in clinical VRET (Multimedia Appendix 1).

### Limitations

The results of this study should be interpreted with several limitations. Apps reviewed were results of a March 2020 search using the term “virtual reality” on the Google Play Store and the Apple App Store. Over the course of the study, 4 Apple App Store apps and 2 Google Play Store apps included during initial screening became unavailable during data extraction. Due to frequent changes in consumer app stores, content analysis studies such as this one should be updated frequently and indefinitely to ensure the most up-to-date insights are available for clinicians. While the use of Web Scraper helped minimize subjective interpretation of apps and human input error, the accuracy of its results may vary depending on the specific web scraper technology used and the technical structure of app store sites. However, web-scraped information in this study was verified by the reviewers prior to data analysis. Another limitation of this study was the focus on smartphone-based VR. Smartphone-based VR was primarily distributed through Google Cardboard and Samsung GearVR, which were discontinued in March 2021 and September 2020, respectively. While smartphone-based VR equipment and software are still functional and for sale, VR consumer markets have shifted toward stand-alone devices such as the Meta Quest 2. Reviewing apps available for the Quest 2 would be important to exploring existing and emerging options for accessible VRET. A final limitation is that apps were characterized by relevant features but not by clinical value. It is uncertain how these results may correspond to clinical validity in the hands of practicing mental health providers. Future research should focus on clinicians’ evaluations to further distill this list of curated VRET apps with direct input of practicing professionals [38].

### Conclusions

We sought to describe the landscape of publicly available smartphone apps with potential clinical utility for VRET by conducting a content analysis of smartphone apps related to VR on the Google Play Store and the Apple App Store. We found that there are 52 apps available on the Google Play Store and 32 on the Apple App Store with elements pertaining to common specific phobias such as bodies of water or weather events, animals, heights, and flying. Most apps were visually abstract and free to use, making them accessible to clinicians and suitable for use as part of exposure hierarchies. However, none of the apps were designed for clinical use, nor do they contain tools for clinician control. Of these available apps, 16 contained features that were ideal to evaluate further, and more research is needed to understand the potential of such apps as part of VR-based exposure therapy.

## Appendix

Multimedia Appendix 1 Supplementary Table 1. Included apps.

## Abbreviations

IVET: in vivo exposure therapy
VR: virtual reality
VRET: virtual reality exposure therapy

## Data Availability

The data sets generated during and/or analyzed during the current study are available from the corresponding author on reasonable request.
