# Supplementary material for: Characterizing Consumer Smartphone Apps for Virtual Reality–Based Exposure Therapy: Content Analysis
Source: J Med Internet Res. 2023 Apr 14;25:e41807. doi: 10.2196/41807 (PMC10148210; doi:10.2196/41807)
Supplement: Multimedia Appendix 1 [file jmir_v25i1e41807_app1.docx]

| Supplementary Table 1. Included apps. | | | | | | |
| --- | --- | --- | --- | --- | --- | --- |
| **Apple App Store** | | | | | | |
| **App Name** | **Phobias Depicted** | **Realism** | **Cost** | **Rating** | **Number of Raters** | **Last Updated** |
| Aquarium VR | Water, Animals | Mixed | Free | 3.2 | 92 | 11/10/2015 |
| Cedar Point VR | Heights | Photorealistic | Free | 3.6 | 83 | 9/16/2015 |
| Colorado VR - Explore Colorado in Virtual Reality | Water | Photorealistic | Free | 3.0 | 3 | 2/1/2017 |
| Dinosaur Battle Virtual Reality - Jurassic VR Game | Animals | Abstract | $1.99 | 0 | 0 | 2/13/2021 |
| Economist VR | Variety | Photorealistic | Free | 5.0 | 2 | 7/9/2019 |
| EON Experience VR | Variety | Mixed | Free | 2.8 | 6 | 6/15/2015 |
| Georgia Tech College of Computing Virtual Reality | Enclosed Spaces | Photorealistic | Free | N/A | 0 | N/A |
| Google Cardboard | Variety | Mixed | Free | 2.9 | 15 | 2/4/2022 |
| Jurassic Virtual Reality (VR) | Animals | Mixed | Free | 3.4 | 153 | 2/14/2021 |
| Magi Chapel VR | Enclosed Spaces | Photorealistic | Free | 5.0 | 3 | 11/10/2015 |
| Mallorca 360° Virtual Reality Experience | Water | Photorealistic | $1.99 | 1.0 | 2 | 7/25/2017 |
| Nebula Virtual Reality Galaxy | Flying | Photorealistic | $0.99 | 1.0 | 1 | 3/19/2021 |
| Roller Coaster VR | Heights | Abstract | Free to try | 3.2 | 118 | 12/20/2018 |
| Roller Coaster VR Theme Park | Heights | Abstract | Free to try | 4.4 | 21400 | 3/16/2022 |
| Serene VR | Water | Photorealistic | Free | 3.9 | 9 | 10/7/2016 |
| Skydive360 | Heights | Abstract | Free | 2.8 | 13 | 2/11/2016 |
| SOAR Virtual Reality | Flying | Photorealistic | Free to try | 3.0 | 3 | 3/6/2018 |
| Survival Dino: Virtual Reality | Animals | Mixed | Free to try | 3.5 | 529 | 7/3/2018 |
| The Walk VR | Heights | Photorealistic | Free | 2.3 | 257 | 10/15/2016 |
| Trail World VR Virtual Reality | Heights, Flying, Water | Abstract | Free | 3.8 | 411 | 10/26/2017 |
| Virtual Reality MRI | Medical | Mixed | Free | N/A | 0 | 8/1/2017 |
| Virtual Reality VR Site Trip | Enclosed Spaces | Photorealistic | Free to try | N/A | 0 | 1/11/2022 |
| VR - Virtual reality Videos | Animal, Water | Mixed | Free to try | 3.4 | 5200 | 5/26/2021 |
| VR Fly With A Real Bald Eagle Virtual Reality 360 | Heights, Animals | Photorealistic | $1.99 | N/A | 0 | 2/14/2017 |
| VR Flying Car Flight Simulator – The best game for google cardboard Virtual Reality | Flying | Abstract | Free | 2.1 | 13 | 7/19/2016 |
| VR Games Pack - All in One 3D Ultimate Experience | Heights | Mixed | $4.99 | 1.0 | 1 | N/A |
| VR Paris Boat Trip - Virtual Reality 360 France | Water | Photorealistic | $1.99 | 1.0 | 1 | 9/23/2016 |
| VR Paris High Up On Eiffel Tower Virtual Reality | Heights | Photorealistic | $1.99 | N/A | 0 | 3/23/2017 |
| VR Paris Palace of Versailles Virtual Reality Tour | Enclosed Spaces | Photorealistic | $1.99 | 1.0 | 1 | 6/9/2017 |
| VR Scuba Diving with Google Cardboard | Water, Animals | Photorealistic | Free to try | 1.6 | 39 | 1/10/2017 |
| VR Showcase | Water | Photorealistic | Free | 3.5 | 4 | 1/12/2017 |
| YouVisit VR - Virtual Reality | Animals | Photorealistic | Free | 3.4 | 25 | 12/22/2016 |
| **Google Play Store** | | | | | | |
| **App Name** | **Phobias Depicted** | **Realism** | **Cost** | **Rating** | **Number of Raters** | **Last updated** |
| Amusement Island VR Cardboard | Variety | Abstract | Free | 3.5 | 137 | 4/23/2020 |
| Astronaut VR | Flying | Abstract | $1.99 | 5.0 | 2 | 1/31/2018 |
| CatHotel VR: Fur-tual Reality | Animals | Abstract | $0.99 | 2.0 | 2 | 2/22/2017 |
| DK Virtual Reality | Variety | Photorealistic | Free | 2.4 | 136 | 6/18/2021 |
| Eagle Survival VR Sim | Flying, Animals | Mixed | Free | 4.6 | 4917 | 9/29/2018 |
| Epic Roller Coasters | Heights | Photorealistic | Free | 3.7 | 553 | 12/25/2018 |
| Falling VR Simulator | Heights | Abstract | Free | 2.3 | 4163 | 12/26/2019 |
| Froggy VR | Animals | Abstract | Free | 3.3 | 1249 | 4/11/2017 |
| PI VR Human Body | Medical | Abstract | Free | 3.3 | 46 | 10/11/2019 |
| Relax River VR | Water | Abstract | Free | 4.5 | 3210 | 1/10/2022 |
| Roller Coaster VR: Ultimate Free Fun Ride | Heights | Mixed | Free | 3.5 | 20908 | 9/26/2019 |
| Rollercoaster VR | Heights | Abstract | $0.99 | 1.7 | 46 | 3/7/2017 |
| RollerCoasterVR DarkCity | Heights | Abstract | Free | 4.1 | 1001 | 4/21/2016 |
| Shark VR sharks games for VR | Animals, Water | Abstract | Free | 3.0 | 7071 | 7/30/2021 |
| Sherlock VR | Enclosed spaces | Abstract | $1.99 | 3.1 | 19 | 12/14/2016 |
| Skydiving Virtual Reality 360º | Heights | Photorealistic | Free | 2.3 | 257 | 11/21/2020 |
| Solar Space Exploration VR Virtual Reality | Flying | Abstract | Free | 3.6 | 267 | 10/25/2019 |
| Stone Age Snap VR | Animals | Abstract | Free | 3.0 | 9 | 11/7/2015 |
| Swim Sharks In Cage VR Simulator | Animals, Water | Mixed | Free | 2.7 | 2830 | 6/9/2018 |
| The Walk VR \| Beautiful jungle World | Water | Photorealistic | Free | 2.7 | 57 | 1/31/2019 |
| Trail World VR Virtual Reality | Variety | Abstract | Free | 3.8 | 945 | 9/27/2018 |
| Treasure Island: Free VR Game | Water | Abstract | Free | 2.7 | 6 | 4/26/2018 |
| Underwater Adventure VR | Water, Animals | Abstract | Free | 2.9 | 486 | 1/13/2022 |
| Underwater VR | Water, Animals | Abstract | Free to try | 3.1 | 435 | 3/24/2019 |
| Voxel Fly | Flying | Abstract | Free to try | 4.4 | 1867 | 12/19/2018 |
| VR Abyss: Sharks & Sea Worlds in Virtual Reality | Water, Animals | Mixed | Free to try | 3.3 | 12739 | 2/20/2021 |
| VR Aqua Thrills | Water | Abstract | Free | 3.0 | 217 | 1/7/2020 |
| VR Christmas | Enclosed spaces | Abstract | $1.49 | N/A | 0 | 12/31/2019 |
| VR City View Rope Crossing - VR Box App | Heights | Abstract | Free to try | 3.5 | 1022 | 6/15/2021 |
| VR Crazy Swing | Heights | Abstract | Free to try | 3.3 | 8999 | 4/28/2017 |
| VR Deep Ocean Roller Coaster (Google Cardboard) | Water | Abstract | Free | 2.8 | 107 | 11/8/2020 |
| VR Diving - Deep Sea Discovery (Cardboard Game) | Water, Animals | Mixed | Free to try | 3.0 | 1074 | 10/12/2018 |
| VR Escape Bird | Flying | Mixed | Free | 3.6 | 347 | 4/12/2020 |
| VR Escape Game | Enclosed spaces | Abstract | Free to try | 3.1 | 1856 | 7/27/2021 |
| VR Falling Tower Dubai | Heights | Abstract | Free | 2.5 | 801 | 7/6/2018 |
| VR Flight Simulator | Flying | Abstract | $0.99 | 2.7 | 3 | 5/14/2017 |
| VR Flight: Airplane Pilot Simulator (Cardboard) | Flying | Abstract | Free | 2.9 | 2239 | 2/7/2019 |
| VR Heights Phobia | Heights | Abstract | Free | 3.1 | 5515 | 12/3/2021 |
| VR Mission Leviathan — underwater expedition | Water, Animals | Abstract | Free to try | 3.4 | 1304 | 12/19/2018 |
| VR Ocean Aquarium 3D | Water, Animals | Abstract | Free to try | 3.8 | 965 | 7/10/2020 |
| VR Roller Coaster 360 | Heights | Abstract | Free to try | 3.2 | 254 | 3/16/2022 |
| VR Roller Coaster Crazy Rider & Adventure Thrills | Heights | Abstract | Free to try | 3.5 | 7220 | 3/4/2022 |
| VR Roller Coaster Sunset - 360 HD simulator | Heights | Abstract | Free to try | 3.9 | 684 | 10/19/2018 |
| VR Safari - Google Cardboard Game | Animals | Abstract | Free | 2.8 | 604 | 9/11/2017 |
| VR Space Stalker | Flying | Abstract | Free to try | 3.4 | 3439 | 6/21/2017 |
| VR Temple Roller Coaster for Cardboard VR | Variety | Abstract | Free to try | 3.4 | 36424 | 2/21/2021 |
| VR Thrills: Roller Coaster 360 (Cardboard Game) | Heights | Mixed | Free to try | 3.5 | 49233 | 2/14/2020 |
| VR Virtual Zoo 3D | Animals | Abstract | Free | 2.9 | 210 | 2/7/2019 |
| VR Zoo - Cardboard | Animals | Mixed | Free | 3.0 | 67 | 10/3/2016 |
| Walk The Plank VR | Heights | Abstract | Free | 3.0 | 5084 | 8/21/2021 |
| Water Ride VR | Water | Abstract | Free | 4.5 | 1407 | 2/20/2020 |
| Waterfall Hunting VR Cardboard | Animals, Water | Photorealistic | Free | 3.0 | 39 | 11/4/2016 |
